# Determinants of Participation and Detection Rate of Colorectal Cancer From a Population-Based Screening Program in China

**DOI:** 10.3389/fonc.2020.01173

**Published:** 2020-07-31

**Authors:** Jiangong Zhang, Huifang Xu, Liyang Zheng, Juan Yu, Qiong Chen, Xiaoqin Cao, Shuzheng Liu, Maria Jose Gonzalez, Lanwei Guo, Xibin Sun, Shaokai Zhang, Youlin Qiao

**Affiliations:** ^1^Department of Cancer Epidemiology and Prevention, The Affiliated Cancer Hospital of Zhengzhou University, Henan Cancer Hospital, Zhengzhou, China; ^2^Endoscopic Diagnosis and Treatment Center, The Affiliated Cancer Hospital of Zhengzhou University, Henan Cancer Hospital, Zhengzhou, China; ^3^School of Public Health, Dalian Medical University, Dalian, China; ^4^Office of Cancer Screening, National Cancer Center/National Clinical Research Center for Cancer/Cancer Hospital, Chinese Academy of Medical Sciences and Peking Union Medical College, Beijing, China

**Keywords:** adherence, colorectal cancer, lesion, early detection, colonoscopy

## Abstract

Colorectal cancer (CRC) screening has been widely implemented in Europe and the USA. However, there is little evidence of participation and diagnostic yields in population-based CRC screening in China. The participation rate and detection of colorectal lesions in this program were reported and related factors were explored. The analysis was conducted in the context of the Cancer Screening Program in Urban China, which recruited 282,377 eligible participants aged 40–74 years from eight cities in Henan province from 2013 to 2019. A total of 39,834 participants were evaluated to be high risk for CRC by an established risk score system and were subsequently recommended for colonoscopy. Of 39,834 with high risk for CRC, 7,454 subjects undertook colonoscopy (participation rate of 18.71%). We found that 50–64 years, high level of education, marriage, former smoking, current alcohol drinking, low levels dietary intake of vegetables, high levels dietary intake of processed meat, lack of physical activity, fecal occult blood test positive result, history of colonic polyp, history of colorectitis, and family history of CRC were associated with increased participation of colonoscopy screening. Overall, 17 CRC (0.23%), 95 advanced adenoma (1.27%), 478 non-advanced adenomas dysplasia (6.41%), 248 hyperplastic polyp (3.33%), and 910 other benign lesions (12.21%) were detected. The findings from the study will provide important references for designing effective population-based CRC screening strategies in the future. Given the relatively low participation rate, there was room for improvement in the yield of CRC screening.

## Introduction

Colorectal cancer (CRC) is one of the most commonly diagnosed cancers worldwide, with an age-standardized incidence rate of 19.4 per 100,000 and an age-standardized mortality rate of 8.9 per 100,000, in 2018 (1). In recent years, the incidence of CRC is increasing in China owing to the improvement of living standards, lifestyle changes, and the growing number of elderly population (2). In China, the world standardized incidence and mortality of CRC were 19.4 and 9.0 per 100,000, respectively. A total of 521,490 new cases and 247,563 deaths were estimated in 2018 in China, which accounted for 28.2 and 28.1% of all the world cases (1).

More than 90% CRCs are developed from colonic polyps, especially adenomatous polyps (3). Studies have shown that it takes about 7–12 years to progress from adenomatous polyps to early CRC. If the treatment is performed at the stage of adenomatous polyps, it can be completely cured and prevent canceration, and the 5-year survival rate can exceed 90% (4). However, the 5-year survival rate for advanced cases is <10% (4). Colonoscopy with biopsies for histologic confirmation has been shown to significantly reduce CRC mortality through early detection of cancer or removal of adenomatous polyps (5). However, colonoscopy is an invasive procedure requiring a high level of expertise, with a high cost (6, 7). In countries with moderate or low CRC incidence and limited medical resources, it is recommended to use a risk stratification scoring system to select high-risk patients for colonoscopy (8, 9). However, there is still evidence that in population-based screening programs, the strategy of combining risk stratification with subsequent colonoscopy remains ineffective. Since October 2012, the Chinese government has launched a population-based Cancer Screening Program in Urban China (CanSPUC). Except for CRC, lung cancer, female breast cancer, liver cancer, esophageal cancer, and gastric cancer were all targeted (10). Henan started this program in 2013. Qualified participants were recruited from the community in the study area and were invited to undergo cancer screening for free. Participants are first invited to conduct cancer risk assessment through the established Clinical Cancer Risk Scoring System. It is recommended that participants who are assessed as high risk for specific types of cancer undergo appropriate screening interventions in accordance with the research protocol. For CRC screening, it is recommended that people at high risk of CRC follow a procedure and go to a designated tertiary hospital for colonoscopy.

In this study, we report the CRC screening results conducted in the first 6 years (from 2013 to 2019) of the program in Henan Province of China. Our aim is to provide evidence of colonoscopy participation and diagnosis in a timely manner. Research on high-risk populations in China provides an important reference for designing effective CRC screening strategies in the future.

## Methods

### Study Design and Population

We carried out a cross-sectional study within the framework of CanSPUC. CanSPUC is an ongoing national cancer screening program launched in October 2013 in Henan province of China. Study methods have been described elsewhere (10). In short, social media and community advertising were used to raise public awareness about the cancer screening process. After then, trained staff provided convocation and appointment services to residents aged 40–74 years who lived in selected communities of participating cities by phone and personal contact. All qualified participants (40–74 years) were interviewed to collect information about their exposure to risk factors by a trained staff, and after signing a written informed consent, their cancer risk was measured using a defined Clinical Cancer Risk Score System. To improve the detection rate of CRC and optimize the use of limited medical resources in this screening program, it is recommended that only participants who are assessed as high risk of CRC to undergo colonoscopy in a tertiary hospital are designated and free of charge. All participants provided a written informed consent, and the study was approved by the Ethics Committee of National Cancer Center/Cancer Hospital, Chinese Academy of Medical Sciences, and Peking Union Medical College.

**Figure 1:**
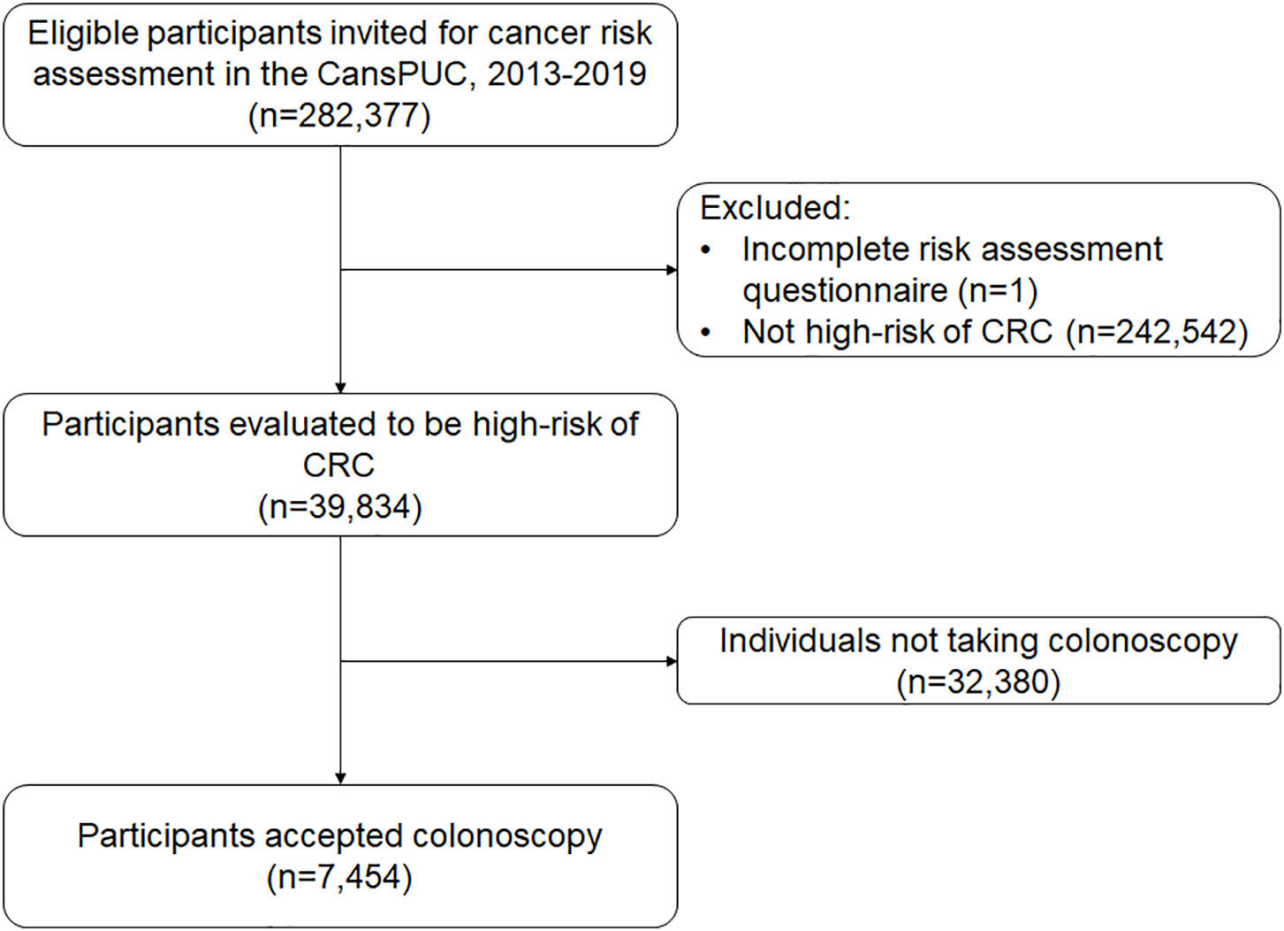
Flow diagram of participant recruitment in CanSPUC, 2013–2019.

In this analysis, we used data from the first 6 years of the CRC screening program in Henan Province, from October 2013 to October 2019. This province covers eight cities (Zhengzhou, Zhumadian, Anyang, Luoyang, Nanyang, Jiaozuo, Puyang, and Xinxiang). Overall, 282,377 eligible participants were recruited. After excluding non-CRC high-risk groups (*N* = 242,542) and participants with ineffective risk assessment results (*N* = 1), the study included 39,834 remaining participants. Figure 1 shows the flowchart for recruiting the population sample of the study.

### Risk Assessment

Participants were asked to perform a risk assessment before performing a colonoscopy. The basic principle of the development of the cancer risk scoring system is based on the Harvard Risk Index (11), but it also includes risk factors, relative risks, and exposure rates of risk factors for the Chinese population. Smoking (at least one cigarette a day for more than 6 months), alcohol drinking (at least once a week for more than 6 months), tea drinking (at least 3 times a week for more than 6 months), dietary intake of pickled food, hot drink or hot food diet, high-salt diet, more-dry diet, body mass index (BMI), indoor soot exposure in the past 10 years, history of intestinal polyps, history of chronic colorectitis, and family history of CRC in first-degree relatives are included in the risk scoring system. The panel of experts assigned each risk factor a score based on the degree of association with CRC. The cumulative risk score was calculated and then divided by the average risk score in the general population to get the final individual relative risk. Individuals whose relative risk exceeds 1.50 were defined as high risk of CRC.

### Clinical Procedures

All colonoscopy tests were performed in a total of nine tertiary hospitals (one in each city, except for two in Zhengzhou) by experienced gastroenterologists (physicians with at least 5 years of experience in performing colonoscopy). The abnormal findings found during the colonoscopy were carefully examined in accordance with standard clinical procedures, and biopsy samples were collected for further pathological diagnosis. Any findings during the colonoscopy were recorded in photographs. Clinical information such as morphological features, macroscopic diagnosis, and size were collected and recorded in a data system.

### Outcome Ascertainment and Quality Control

Pathological examination was used for all abnormal findings found during colonoscopy by following the latest clinical guidelines. Pathological results were collected from highly standardized forms filled in by pathologists. For difficult cases with difficult or uncertain pathological diagnosis, the expert team of the National Cancer Center of China conducted consultation, and the report of the consultation results was forwarded to the respective doctors.

In this study, advanced adenomas are defined as at least one adenoma with villous components or at least one adenoma ≥10 mm or high-grade dysplasia.

### Data Acquisition

Trained staff and physicians collect standardized paper documents (epidemiological questionnaires, colonoscopy reports, and pathology reports). Trained study staff checked the validity of forms and entered it into the data management system. If an inconsistency was found during the consistency check, the error was corrected by retrieving the original record. A unique identifier was used for each participant to track all relevant document forms of the individual. All data were transferred to the central data management team from the National Cancer Center of China, where the database was established and analyzed.

### Statistical Analysis

In addition to the descriptive analysis of the characteristics of the study population, the overall participation rate and specific group participation rate owing to public factors were calculated, and a 95% CI was reported by Clopper–Pearson exact test. Chi-square test was used to compare differences in participation rates between groups. Associations between factors, including age (categorized into 40–44, 45–49, 50–54, 55–59, 60–64, 65–69, and 70–74 years), sex (male, female), BMI (<18.5, 18.5–24.0, 24.0–28.0, and ≥28.0 kg/m^2^), waist (male: <85 and ≥85 cm; female: <80 and ≥80 cm), marriage status (unmarried or divorce or widowed, married), educational background (primary school or below, junior or senior high school, undergraduate or over), smoking status (never, current, former), alcohol drinking (never, current, former), dietary intake of vegetables (<2.5 kg/week, ≥2.5 kg/week), dietary intake of fruit (<1.25 kg/week, ≥1.25 kg/week), dietary intake of processed meat (<0.35 kg/week, ≥0.35 kg/week), physical activity (<3 times/week, ≥3 times/week), past fecal occult blood test (FOBT) (no, negative result, positive result, unknown result), history of colonic polyp (no, yes), history of colorectitis (no, yes), family history of CRC (no, yes), and colonoscopy participation rate were quantified by non-conditional logistic regression models and two-level logistic regression models with ORs and their 95% CIs. For two-level logistic regression model, the first level was the individual level (age, sex, BMI, waist, education background, marriage, smoking, alcohol drinking, dietary intake of vegetable, dietary intake of fruit, dietary intake of processed meat, physical activity, past FOBT, history of colonic polyp, history of colorectitis, and family history of CRC) and the second level was the geographical level (study sites). The diagnostic rate of colonoscopy was calculated, including the detection of CRC, and the detection rate of age and gender. The yield per 10,000 invitees and the number of colonoscopy used to detect a colorectal lesion were also calculated. Statistical software SAS version 9.4 (SAS Institute, Cary, NC) and STATA 14.0 were used for all statistical analyses. All tests are double-sided tests, and *p* ≤ 0.05 are considered statistically significant.

## Results

### Characteristics of the Study Population and Participation Rates

Table 1 lists the characteristics of people at high risk of CRC. Overall, more women (55.06%) were included in the study. The average age was 55.44 ± 8.36 years, and most (72.17%) were between 45 and 64 years old. About 63% of the participants (*N* = 24,987) were overweight or obese, and about 65% of them (*N* = 25,947) had abdominal obesity (present with waist). About 65% of the participants (*N* = 25,883) graduated from junior/senior high school, and most of them (96%, *N* = 38,255) were married. About 38% of the participants (*N* = 15,111) were current smokers or past smokers, and about 42% of them (*N* = 16,744) are current or past alcohol drinkers. About 66% of the participants (*N* = 26,221) had a dietary intake of vegetables with <2.5 kg/week, and about 73% of them (*N* = 29,004) had a dietary intake of fruit with <1.25 kg/week. About 51% of the participants (*N* = 20,211) had a dietary intake of processed meat with more than 0.35 kg/week, and about 64% of them (*N* = 25,475) participated in physical activity <3 times a week. About 17% of the high-risk population received FOBT previously and 28.48% of them (*N* = 1,981) had positive FOBT results. About 17% of the participants (*N* = 6,919) had a history of colonic polyp, and about 53% of them (*N* = 21,076) had a history of colorectitis. About 17% of the participants (*N* = 6,247) had a family member diagnosed with CRC. Of the 39,834 high-risk CRC participants, 7,454 of them underwent colonoscopy according to our research recommendations. The overall participation rate was 18.71% (95% CI 18.08–18.72%). Overall, the participation rate of men was slightly higher than that of women (19.30 vs. 18.23%, *p* = 0.007). They were also higher among participants in the 45–64 age group. Univariate analyses showed that participants who had a normal BMI (18.5–24.0 kg/m^2^), had a smaller waist, had a high educational background, were unmarried/divorce/widowed, were current or former smokers, were current or former alcohol drinkers, had low levels of dietary intake of vegetables, had low levels of dietary intake of fruit, physical inactivity, had positive FOBT results, had a history of colonic polyp, had a history of colorectitis, or had a family history of CRC had relatively higher participation rates.

**Table 1:** Characteristics of the study population and participation rates between different groups.

**Factors**	**Participants at high risk for CRC (%)**	**Participants undertaking colonoscopy (%)**	**Participation rate (%)**	**χ^2^**	*P-*value*
**Age (years)**				98.74	<0.001
40–44	4,255 (10.68)	760 (10.20)	17.86		
45–49	6,982 (17.53)	1,317 (17.67)	18.86		
50–54	8,011 (20.11)	1,636 (21.95)	20.42		
55–59	6,714 (16.85)	1,372 (18.41)	20.43		
60–64	7,044 (17.68)	1,331 (17.86)	18.90		
65–69	5,113 (12.84)	827 (11.09)	16.17		
70–74	1,715 (4.31)	211 (2.83)	12.30		
**Sex**				7.39	0.007
Male	17,901 (44.94)	3,455 (46.35)	19.30		
Female	21,933 (55.06)	3,999 (53.65)	18.23		
**BMI (kg/m**^**2**^**)**				30.46	<0.001
<18.5	533 (1.34)	99 (1.33)	18.57		
18.5–24.0	14,314 (35.93)	2,814 (37.75)	19.66		
24.0–28.0	17,175 (43.12)	3,240 (43.47)	18.86		
≥28.0	7,812 (19.61)	1,301 (17.45)	16.65		
**Waist (cm)**				6.75	0.009
<85 (male)/ <80 (female)	13,887 (34.86)	2,695 (36.16)	19.41		
≥85 (male)/≥80 (female)	25,947 (65.14)	4,759 (63.84)	18.34		
**Educational background**				219.28	<0.001
Primary school or below	6,176 (15.50)	940 (12.61)	15.22		
Junior/senior high school	25,883 (64.98)	4,627 (62.07)	17.88		
Undergraduate or over	7,775 (19.52)	1,887 (25.32)	24.27		
**Marriage**				10.21	0.001
Unmarried/divorce/widowed	1,579 (3.96)	344 (4.61)	21.79		
Married	38,255 (96.04)	7,110 (95.39)	18.59		
**Smoking**				34.38	<0.001
Never	24,723 (62.07)	4,479 (60.09)	18.12		
Current	12,262 (30.78)	2,332 (31.29)	19.02		
Former	2,849 (7.15)	643 (8.63)	22.57		
**Alcohol drinking**				59.91	<0.001
Never	23,090 (57.97)	4,027 (54.02)	17.44		
Current	14,607 (36.67)	3,010 (40.38)	20.61		
Former	2,137 (5.36)	417 (5.59)	19.51		
**Dietary intake of vegetables**				65.31	<0.001
<2.5 kg/week	26,221 (65.83)	5,205 (69.83)	19.85		
≥2.5 kg/week	13,613 (34.17)	2,249 (30.17)	16.52		
**Dietary intake of fruit**				45.88	<0.001
<1.25 kg/week	29,004 (72.81)	5,662 (75.96)	19.52		
≥1.25 kg/week	10,830 (27.19)	1,792 (24.04)	16.55		
**Dietary intake of processed meat**				0	0.959
<0.35 kg/week	19,623 (49.26)	3,674 (49.29)	18.72		
≥0.35 kg/week	20,211 (50.74)	3,780 (50.71)	18.70		
**Physical activity**				33.70	<0.001
<3 times/week	25,475 (63.95)	4,984 (66.86)	19.56		
≥3 times/week	14,359 (36.05)	2,470 (33.14)	17.20		
**Past FOBT**				54.76	<0.001
No	32,879 (82.54)	6,071 (81.45)	18.46		
Yes (negative result)	3,379 (8.48)	580 (7.78)	17.16		
Yes (positive result)	1,981 (4.97)	490 (6.57)	24.73		
Yes (unknown result)	1,595 (4.00)	313 (4.20)	19.62		
**History of colonic polyp**				438.14	<0.001
No	32,915 (82.63)	5,542 (74.35)	16.84		
Yes	6,919 (17.37)	1,912 (25.65)	27.63		
**History of colorectitis**				303.71	<0.001
No	18,758 (47.09)	2,833 (38.01)	15.10		
Yes	21,076 (52.91)	4,621 (61.99)	21.93		
**Family history of CRC**				585.68	<0.001
No	33,587 (84.32)	5,600 (75.13)	16.67		
Yes	6,247 (15.68)	1,854 (24.87)	29.68		

*CRC, colorectal cancer; BMI, body mass index; FOBT, fecal occult blood test*.

### Multivariable Analysis for Factors Related to Participation Rate

As shown in Table 2, we also conducted a multivariable logistic regression model and a two-level logistic regression model to explore potential factors related to the participation rate. After adjusting for potential influencing factors, we found that age, education background, marriage, smoking, alcohol drinking, dietary intake of vegetables, dietary intake of processed meat, physical activity, FOBT results, history of colonic polyp, history of colorectitis, and family history of CRC were associated with participation rate. For instance, the odds of a participant with a history of colonic polyp undertaking screening colonoscopy was 0.53-fold higher than for a participant with no history of colonic polyp (OR 1.53, 95% CI 1.43–1.63). Similarly, the odds of a participant with a family history of CRC undertaking screening colonoscopy was 0.69-fold higher than for a participant with no family history of CRC (OR 1.69, 95% CI 1.58–1.81). After changing to the two-level logistic regression model in model II, the respective ORs did not change much (Table 2).

**Table 2:** Odds ratios of factors associated with participation rate of colonoscopy in the screening program.

**Factors**	**Model I**^****§****^		**Model II**^****¶****^	
	**Odds ratio (95% CI)**	***p*-value**	**Odds ratio (95% CI)**	***p*-value**
**Age (years)**				
40–44	1.00		1.00	
45–49	1.09 (0.99–1.21)	0.094	1.09 (0.99–1.21)	0.092
50–54	1.25 (1.14–1.38)	<0.001	1.25 (1.14–1.38)	<0.001
55–59	1.30 (1.17–1.44)	<0.001	1.30 (1.17–1.44)	<0.001
60–64	1.20 (1.08–1.33)	0.001	1.20 (1.08–1.33)	0.001
65–69	1.03 (0.92–1.15)	0.624	1.03 (0.92–1.16)	0.611
70–74	0.73 (0.61–0.86)	<0.001	0.73 (0.61–0.86)	<0.001
**Sex**				
Male	1.00		1.00	
Female	0.95 (0.88–1.02)	0.171	0.95 (0.88–1.02)	0.178
**BMI (kg/m**^**2**^**)**				
<18.5	1.00		1.00	
18.5–24.0	1.03 (0.82–1.29)	0.806	1.03 (0.82–1.29)	0.806
24.0–28.0	1.00 (0.80–1.26)	0.984	1.00 (0.80–1.26)	0.983
≥28.0	1.01 (0.79–1.28)	0.961	1.01 (0.79–1.28)	0.958
**Waist (cm)**				
<85 (male)/ <80 (female)	1.00		1.00	
≥85 (male)/≥80 (female)	0.98 (0.92–1.05)	0.578	0.98 (0.92–1.05)	0.575
**Educational background**				
Primary school or below	1.00		1.00	
Junior/senior high school	1.05 (0.97–1.14)	0.200	1.06 (0.97–1.14)	0.190
Undergraduate or over	1.45 (1.32–1.59)	<0.001	1.45 (1.32–1.59)	<0.001
**Marriage**				
Unmarried/divorce/widowed	1.00		1.00	
Married	0.83 (0.73–0.94)	0.003	0.83 (0.73–0.94)	0.003
**Smoking**				
Never	1.00		1.00	
Current	0.84 (0.78–0.92)	<0.001	0.84 (0.78–0.92)	<0.001
Former	1.20 (1.07–1.34)	0.002	1.20 (1.07–1.34)	0.002
**Alcohol drinking**				
Never	1.00		1.00	
Current	1.13 (1.05–1.21)	0.001	1.13 (1.05–1.21)	0.001
Former	1.04 (0.92–1.17)	0.558	1.04 (0.92–1.17)	0.553
**Dietary intake of vegetables**				
<2.5 kg/week	1.00		1.00	
≥2.5 kg/week	0.88 (0.82–0.94)	<0.001	0.88 (0.82–0.94)	<0.001
**Dietary intake of fruit**				
<1.25 kg/week	1.00		1.00	
≥1.25 kg/week	0.98 (0.91–1.05)	0.523	0.98 (0.91–1.05)	0.509
**Dietary intake of processed meat**				
<0.35 kg/week	1.00		1.00	
≥0.35 kg/week	1.12 (1.06–1.18)	<0.001	1.12 (1.06–1.18)	<0.001
**Physical activity**				
<3 times/week	1.08 (1.02–1.15)	0.007	1.08 (1.02–1.14)	0.008
≥3 times/week	1.00		1.00	
**Past fecal occult blood test**				
No	1.00		1.00	
Yes (negative result)	0.91 (0.82–1.00)	0.055	0.91 (0.83–1.00)	0.058
Yes (positive result)	1.24 (1.11–1.38)	<0.001	1.24 (1.11–1.38)	<0.001
Yes (unknown result)	0.99 (0.87–1.13)	0.915	0.99 (0.87–1.13)	0.915
**History of colonic polyp**				
No	1.00		1.00	
Yes	1.53 (1.43–1.63)	<0.001	1.53 (1.43–1.63)	<0.001
**History of colorectitis**				
No	1.00		1.00	
Yes	1.58 (1.50–1.67)	<0.001	1.58 (1.49–1.67)	<0.001
**Family history of CRC**				
No	1.00		1.00	
Yes	1.69 (1.58–1.81)	<0.001	1.69 (1.58–1.80)	<0.001

§ *Odds ratios were adjusted for factors including study sites, age, sex, BMI, waist, education background, marriage, smoking, alcohol drinking, dietary intake of vegetable, dietary intake of fruit, dietary intake of processed meat, physical activity, past FOBT, history of colonic polyp, history of colorectitis, and family history of CRC in the non-conditional logistic regression model*.

¶ *The model included the individual level (age, sex, BMI, waist, education background, marriage, smoking, alcohol drinking, dietary intake of vegetable, dietary intake of fruit, dietary intake of processed meat, physical activity, past FOBT, history of colonic polyp, history of colorectitis, and family history of CRC) and the geographical level (study sites). All models were controlled for year of recruitment*.

### Colorectal Findings Under Screening Colonoscopy

Table 3 presents the diagnostic yield of colonoscopy in our screening program. Overall, there were 17 CRC, 95 advanced adenoma, 478 non-advanced adenomas dysplasia, 248 hyperplastic polyp, and 910 other benign lesions. This yielded the detection rates for CRC, advanced adenoma, non-advanced adenomas dysplasia, hyperplastic polyp, and other benign lesions at 0.23, 1.27, 6.41, 3.33, and 12.21%, respectively. In addition, based on the gender and age-adjusted detection rate for the Chinese standard population (1982), we calculated the number of colonoscopy tests required to detect one CRC, one advanced adenoma, one non-advanced adenomas dysplasia, one hyperplastic polyp, and one other benign lesions as 1840.7, 365.9, 71.3, 127.6, and 34.7, respectively. In terms of diagnostic yield per invitee, 4.3 CRC, 23.8 advanced adenomas, 119.9 non-advanced adenomas, 62.2 hyperplastic polyp, and 228.4 other benign lesions could be detected per 10,000 invitees.

**Table 3:** Colonic lesions detected by colonoscopy in the screening program.

**Findings**	**Participants taking screening colonoscopy (%)**	**Yield per 10,000 invitees**	**Number of colonoscopies to detect one lesion^&^**
Colorectal cancer	17 (0.23)	4.3	1,840.7
Advanced adenoma	95 (1.27)	23.8	365.9
≥10 mm	34 (0.46)	8.5	1,002.1
Non-advanced adenomas dysplasia	478 (6.41)	119.9	71.3
Hyperplastic polyp	248 (3.33)	62.2	127.6
Other benign lesions	910 (12.21)	228.4	34.7

& *Calculation was based on the age-specific and sex-specific detection rate adjusted by China Standard Population (1982)*.

**Figure 2:**
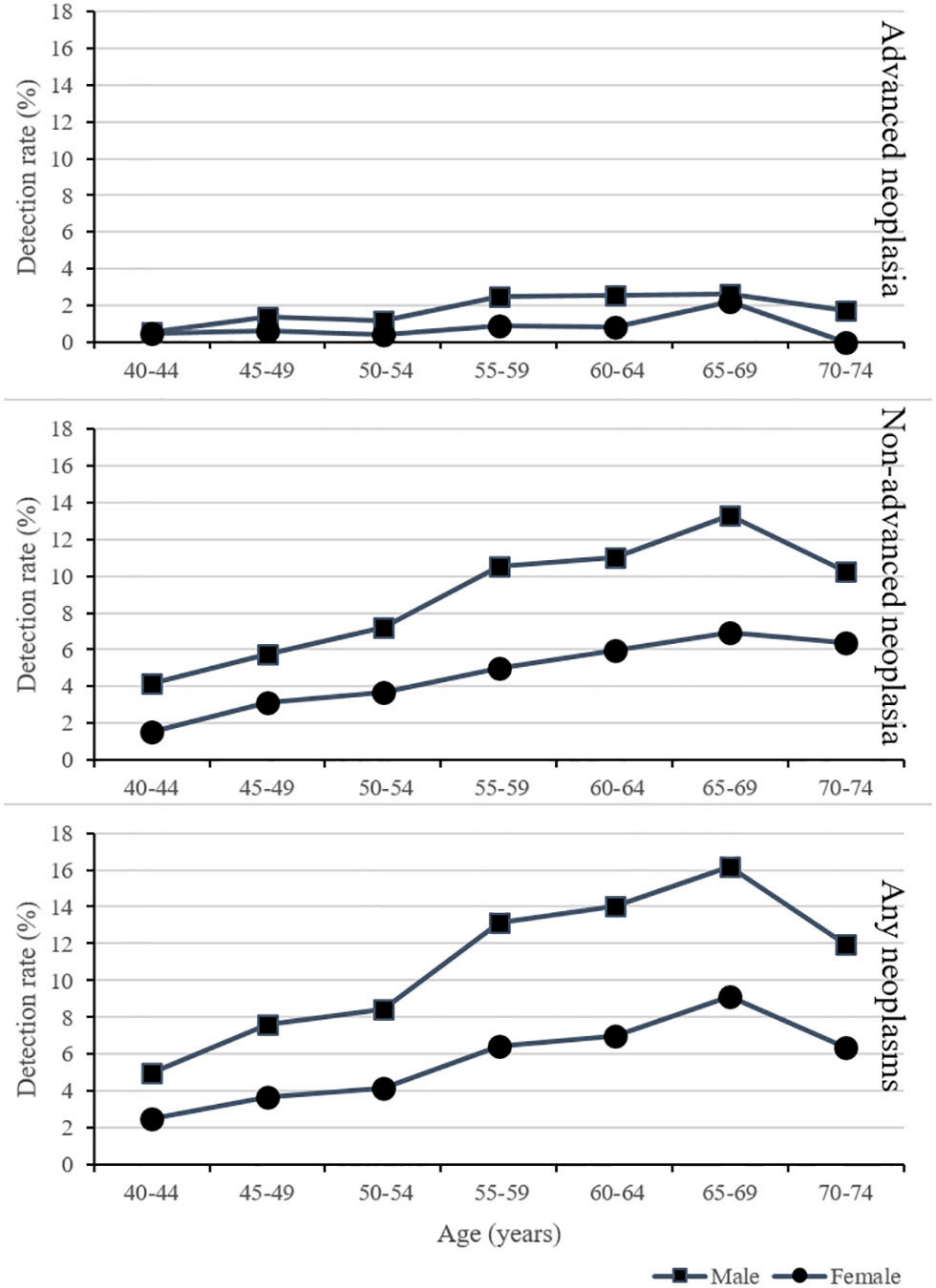
Detection rates of colorectal lesion stratified by age and sex.

As shown in Figure 2, the detection rates for advanced neoplasms, non-advanced adenomas, and any neoplasms increased with age and were higher for male than for female. The detection rate was highest in the group aged 65–69.

## Discussion

This study used CRC screening data obtained in the CanSPUC from 2013 to 2019. The study population covered 40,000 people in eight cities across Henan province. The study found that the overall participation rate of colonoscopy screening for high-risk populations in urban areas was relatively low (18.71%, 95% CI 18.08–18.72%), and there were certain regional differences. In this study, the identification of high-risk populations for colonoscopy screening through the evaluation of a high-risk cancer risk questionnaire is one of the screening strategies recommended in the existing consensus on screening for colorectal cancer in China, which can well find out colorectal cancer and precancerous lesions (12). In Europe and the USA, colonoscopy screening is usually recommended for people at average risk of 50 years and older. Preliminary analysis of a randomized controlled trial (RCT) underway in Europe revealed that the participation rate of colonoscopy screening in four participating countries (Norway, Sweden, Poland, and the Netherlands) was 22.9–60.7% (12). Another RCT in Italy reported a colonoscopy screening participation rate of 24.6% in the average-risk population (13). It can be seen that the poor population compliance of colonoscopy screening is a common problem in many countries.

This study found that people who had previously performed a FOBT test and who tested positive were more likely to undergo colonoscopy screening, which is consistent with findings from other studies (9). For instance, in a RCT conducted in Spain, the participation rates of colonoscopy group and fecal immunochemical test (FIT) group were 34.2 and 24.6%, respectively (*p* < 0.001) (14). A recent study from England reported that the participation rate using FOBT even increased to 66.4% in the National Health Service Bowel Cancer Screening Programme (15). FOBT is one of the common screening methods for colorectal cancer screening recommended by current screening guidelines (16). Compared with colonoscopy, FOBT has higher population compliance and lower cost in population screening (17). In terms of diagnostic ability, the newly developed FIT has shown good diagnostic sensitivity and specificity for CRC, but its diagnostic sensitivity for precancerous lesions is still poor (18). Therefore, how to play the role of FOBT in the organizational screening of the population—both to improve the compliance of the screening and to ensure a higher detection rate of cancer and precancerous lesions—needs to be further explored in future research.

History of colonic polyp, history of colorectitis, and family history of CRC are all important risk factors for CRC that have been confirmed by research (19). This study found that people with these characteristics have better colonoscopy screening compliance. From the clinical point of view, the diagnosis of colonic polyp and colorectitis usually requires colonoscopy to confirm the diagnosis, and doctors will recommend regular colonoscopy review in this high-risk group, and those with a family history of CRC may have a higher recognition of the importance of CRC screening.

This study also found that the participation rate of colonoscopy screening among people aged 40–49 years and with lower education level was lower, which is consistent with the findings of the previous research (20). The analysis may be related to weak health awareness in those groups of people. Therefore, in future screenings, active, and effective health education for these characteristic populations, and raising their awareness of the meaning of CRC screening, will have positive significance for improving compliance with CRC screening.

We also found that the participation rate of colonoscopy screening among people with a history of smoking, alcohol drinking, low level of dietary intake of vegetables, high level of dietary intake of processed meat, and who lack physical activity was higher. A possible explanation to this rate includes that these people may have realized that their unhealthy lifestyle affected their health and thus increase compliance with colonoscopy (9). It needs to be verified in future research.

Through screening, it was found that the detection rate of CRC in urban residents was 0.23%, and the detection rate of advanced adenoma was 1.27%, which is at a low level, lower than the previously reported 0.25 and 3.07% (21). The detection rate in males was higher than in females, which is consistent with the characteristics of CRC incidence in males higher than females (22). With the increase of age, the detection rate of advanced neoplasms, non-advanced adenomas, and any neoplasms increased both for males and females, suggesting that CRC screening is more effective in the elderly population (23). However, the detection rate in the 70–74 age group decreased significantly, which is inconsistent with the trend of CRC in China (24). We noticed that the 70–74 age group had the lowest compliance with colonoscopy (12.30%), and compliance may be an important reason for restricting the detection rate (25). Therefore, improving the screening compliance of the elderly is especially important.

When interpreting our results, we should pay special attention to its strengths and limitations. One of the main advantages of this study is that our analysis comes from a large population-based cancer screening program in China. In addition, well-trained researchers collected detailed patient information in a standardized manner, including epidemiological questionnaires and clinical examination data (colonoscopy and pathology) to ensure data quality. Each year, a team conducts competency training and conducts a centralized review of colonoscopy and pathology reports to improve the consistency and accuracy of clinical diagnosis. However, this study has some limitations. First, the percentage of the population undergoing endoscopic evaluation was very low (less than one-fifth), limiting the implications of the sample to the larger populations. Second, although we selected a population sample from eight cities, our study cannot represent the total population of Henan Province, so selection bias cannot be ruled out. Third, in view of the continuing follow-up of patients diagnosed with CRC, clinical disease information has not been fully obtained. Therefore, our study did not report tumor staging information. Finally, the information, such as on diet, were obtained through a questionnaire survey and not based on nutrition surveys, which might lead to information bias.

## Conclusions

In summary, the participation rate of colonoscopy screening in high-risk urban populations in China is low. Taking effective interventions for subgroups with corresponding characteristics may improve the compliance of CRC screening in future population screening.

## Data Availability Statement

The raw data supporting the conclusions of this article will be made available by the authors, without undue reservation.

## Ethics Statement

The studies involving human participants were reviewed and approved by Ethics Committee of National Cancer Center/Cancer Hospital, Chinese Academy of Medical Sciences, and Peking Union Medical College. The patients/participants provided their written informed consent to participate in this study. Written informed consent was obtained from the individual(s) for the publication of any potentially identifiable images or data included in this article.

## Author Contributions

LG, SZ, and JZ: conception and design. LG and LZ: statistical analyses. LG, HX, MJ, SZ, SL, JY, LZ, QC, XC, and YQ: data acquisition and data interpretation. JZ: drafting of the article. All authors: revised the manuscript and approved the final version of the manuscript.

## Conflict of Interest

The authors declare that the research was conducted in the absence of any commercial or financial relationships that could be construed as a potential conflict of interest.
